# SARS-CoV-2 Nucleocapsid Protein Antagonizes GADD34-Mediated Innate Immune Pathway through Atypical Foci

**DOI:** 10.3390/molecules29204792

**Published:** 2024-10-10

**Authors:** Jie Liu, Guanwen Guan, Chunxiu Wu, Bingbing Wang, Kaifei Chu, Xu Zhang, Su He, Naru Zhang, Geng Yang, Zhigang Jin, Tiejun Zhao

**Affiliations:** 1 Key Laboratory of Novel Targets and Drug Study for Neural Repair of Zhejiang Province, School of Medicine, Hangzhou City University, Hangzhou 310015, China; 2 College of Life Sciences, Zhejiang Normal University, Jinhua 321004, China

**Keywords:** SARS-CoV-2, nucleocapsid protein, GADD34, stress granules, innate immunity

## Abstract

The integrated stress response, especially stress granules (SGs), contributes to host immunity. Typical G3BP1^+^ stress granules (tSGs) are usually formed after virus infection to restrain viral replication and stimulate innate immunity. Recently, several SG-like foci or atypical SGs (aSGs) with proviral function have been found during viral infection. We have shown that the severe acute respiratory syndrome coronavirus 2 (SARS-CoV-2) nucleocapsid (N) protein induces atypical N^+^/G3BP1^+^ foci (N^+^foci), leading to the inhibition of host immunity and facilitation of viral infection. However, the precise mechanism has not been well clarified yet. In this study, we showed that the SARS-CoV-2 N (SARS2-N) protein inhibits dsRNA-induced growth arrest and DNA damage-inducible 34 (GADD34) expression. Mechanistically, the SARS2-N protein promotes the interaction between *GADD34* mRNA and G3BP1, sequestering *GADD34* mRNA into the N^+^foci. Importantly, we found that GADD34 participates in IRF3 nuclear translocation through its KVRF motif and promotes the transcription of downstream interferon genes. The suppression of GADD34 expression by the SARS2-N protein impairs the nuclear localization of IRF3 and compromises the host’s innate immune response, which facilitates viral replication. Taking these findings together, our study revealed a novel mechanism by which the SARS2-N protein antagonized the GADD34-mediated innate immune pathway via induction of N^+^foci. We think this is a critical strategy for viral pathogenesis and has potential therapeutic implications.

## 1. Introduction

Upon the invasion of pathogens such as viruses, the innate immunity system in the host acts as an important defense against viral infection, in which the induction of type I interferon (IFN-I) cytokines (e.g., IFN-α/β) plays a critical role [1]. In the context of RNA virus infections, viral RNA is detected by RIG-I-like receptors (RLRs) [2,3], which interact with the adapter mitochondrial antiviral signaling protein (MAVS, also termed IPS-1) [4]. MAVS serves as a scaffold and recruits the two IKK-related kinases, TANK binding kinase 1 (TBK1) or inducible IκB kinase (IKKi), both of which phosphorylate transcriptional factor IRF3/7 [5]. After phosphorylation and dimerization, IRF3 translocates to the nucleus and activates the transcription of IFN-α/β [6].

Viruses have developed sophisticated strategies to counteract the host’s innate immune defenses and evade immune elimination. Indeed, growing evidence has revealed that SARS-CoV-2 employs a suite of gene-encoded products that impair the host’s innate immune signaling pathways at multiple points and circumvent the IFN-I response. For example, SARS-CoV-2 structural proteins, including nucleocapsid (N), spike (S), and membrane (M), interact with IRF3 [7,8,9], preventing the phosphorylation and nuclear translocation of IRF3. Moreover, some non-structural proteins of SARS-CoV-2, such as Nsp1 [10], Nsp3 [11], Nsp5 [12], Nsp6 [13], and Nsp13 [14], as well as accessory proteins ORF6 and ORF9b [15], have been identified to interfere with the activation of IRF3 by engaging with PPRs. While research has revealed that viral proteins are involved in immune suppression during SARS-CoV-2 infection, the mechanisms by which SARS-CoV-2 antagonizes host immune response remain poorly understood.

In response to viral infection, which is one of the major stressors, viral dsRNA triggers PKR activation and subsequently phosphorylates eIF2α [16,17], thereby inhibiting mRNA translation [18,19] and facilitating the formation of typical stress granules (tSGs) [20,21]. These tSGs, which are membraneless condensates of mRNA–protein complexes, serve as an important host antiviral defense mainly by repressing viral mRNA translation [22]. In addition to translational inhibition of viral mRNA, SGs also engage in the host’s innate immune response [18]. During viral infection, SGs function as platforms that recruit the effectors of the innate immune response, such as RIG-I, leading to efficient activation of IRF3 and production of IFN-I [18]. IFNs activate interferon-stimulated genes (ISGs) that include several effector proteins, such as PKR or 2′-5′-oligoadenylate synthetase 2, which limit viral proliferation and activity within the host cell [23]. Nevertheless, viruses have evolved sophisticated tactics to hinder the assembly of tSGs and evade the host’s antiviral response [24,25,26]. Notably, the induction of SG-like foci or atypical SGs (aSGs) with proviral function has been found during viral infection. For instance, the Picornavirus protease EV71 cleaves eIF4GI, leading to the inhibition of tSGs assembly and the formation of aSGs, which benefit viral translation by sequestering host mRNAs only [27]. Semliki Forest virus [28] and Chikungunya virus [29,30] Nsp2 and Nsp3 proteins recruit G3BP1 into SG-like foci, which engage components of the host translation complex and are beneficial to viral replication. The Rotavirus redirects SG-associated proteins into novel aggregates proximal to viral replication factories, potentially facilitating viral replication and packaging [31]. In our previous study, we found that the SARS2-N protein attenuates PKR activation to inhibit tSGs [32] and hijacks G3BP1 to induce the formation of atypical N^+^/G3BP1^+^ foci (N^+^foci) [33], which in turn suppresses the host’s innate immune response. However, the underlying mechanisms by which N^+^foci suppress innate immunity have not been fully elucidated.

Viral dsRNA triggered two concomitant and conflicting events in host cells. Viral dsRNA-induced phosphorylation of eIF2α inhibits global protein synthesis [17,18] yet viral dsRNA also induces the production of cytokines [1,2,3]. Indeed, both of these are essential for the antiviral response. The accumulation of phosphorylated eIF2α (p-eIF2α) promotes the expression of GADD34 [34,35]. GADD34 interacts with the catalytic subunit of protein phosphatase 1 (PP1) and targets p-eIF2α for dephosphorylation [36], which is crucial for the synthesis of IFN-β during the global suppression of protein translation [37,38,39,40]. The role of GADD34 in response to virus infection has been documented [41,42]. It has been shown that functional GADD34 participates in the RIG-I signaling pathway, which is absolutely required for type I-IFN and IL-6 production in response to dsRNA [42,43]. Intriguingly, we observed that the expression of GADD34 was impaired by the SARS2-N protein. However, the involvement of GADD34 in the cellular immune response to SARS-CoV-2 infection remains to be elucidated.

In this study, we provided insight into a novel mechanism by which SARS-CoV-2 suppresses IRF3 via atypical N^+^/G3BP1^+^ foci (N^+^foci). Mechanistically, the SARS2-N protein promotes the interaction between *GADD34* mRNA and G3BP1 and sequesters *GADD34* mRNA into the N^+^foci, thereby blocking the translation of *GADD34* mRNA. Importantly, we found that expression of the GADD34 protein contributes to the nuclear translocation of IRF3 during dsRNA-induced innate immunity and counteracts the SARS2-N protein’s regulation of innate immune response. Thus, reciprocal inhibition between the SARS2-N protein and GADD34 modulates the innate immune response and viral replication, which may be a critical step during the pathogenesis of SARS-CoV-2 infection and represents a potential target for antiviral therapy.

## 2. Results

### 2.1. SARS-CoV-2 N Protein Inhibits dsRNA-Induced GADD34 Translation

**Figure 1:**
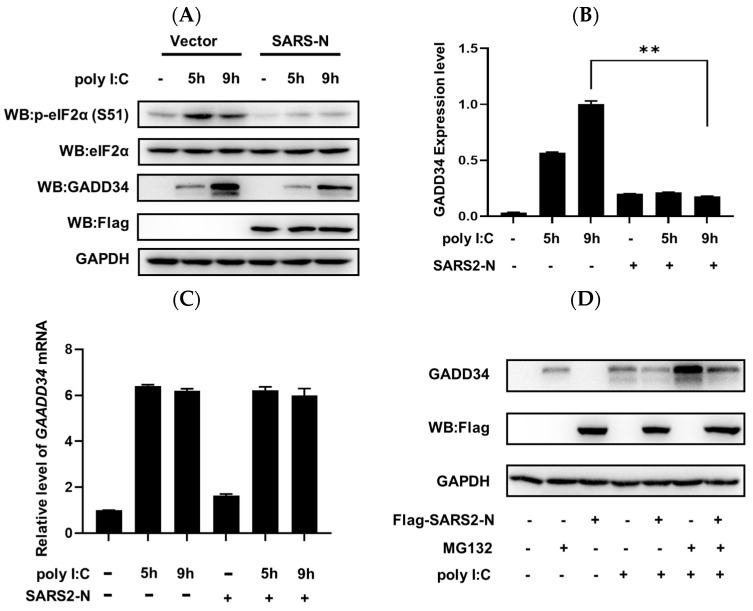

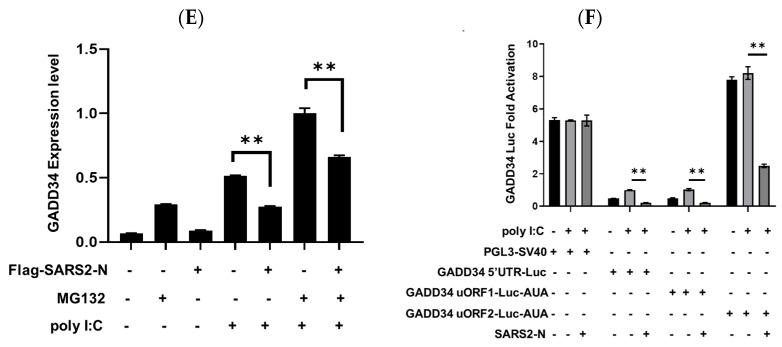
SARS2-N protein inhibits dsRNA-induced GADD34 expression. (**A**) 16HBE cells were transfected with pCS2-Flag-SARS2-N and treated with poly I:C for the indicated time. Cell lysates were subjected to Western blot to detect eIF2α, p-eIF2α (S51), GADD34, FLAG-SARS2-N, and GAPDH. (**B**) Graphical representation of the relative amount of GADD34 to GAPDH shown in panel (**A**). The bars indicate the mean ± SD. Statistics: Student’s *t*-test (**, *p* < 0.01). (**C**) 16HBE cells were transfected with pCS2-Flag-SARS2-N and were untreated or treated with poly I:C for the indicated time. An RT-qPCR experiment was performed to detect the mRNA level of GADD34 and 18S RNA. (**D**) 16HBE cells were transfected with pCS2-Flag-SARS2-N and poly I:C. After treatment with 10 μM of MG132, cell lysates were subjected to Western blot to detect GADD34, FLAG-SARS2-N, and GAPDH. (**E**) Graphical representation of the relative amount of GADD34 to GAPDH is shown in panel D. Bars indicate mean ± SD. Statistics: Student’s *t*-test (**, *p* < 0.01). (**F**) 16HBE cells were transfected with pGL3-SV40, GADD34 5′-UTR, GADD34 uORF1-AUA, or GADD34 uORF2-AUA reporter plasmids (SV40-Luc, GADD34 5′-UTR-Luc, GADD34 uORF1-AUA-Luc, and GADD34 uORF2-AUA-Luc) and expression vectors for SARS2-N (pCS2-Flag-SARS2-N) After treating the cells with poly I:C for 9 h, a dual-luciferase assay was performed. Statistics: Student’s *t*-test (**, *p* < 0.01).

We previously reported that the SARS-CoV-2 N (SARS2-N) protein inhibits innate immune response by remodeling tSGs to N^+^foci [33]. Here we tried to further investigate the mechanism underlying the suppression of innate immunity by N^+^foci. First, we found that the treatment with poly I:C, which is a synthetic analog of viral dsRNA, could induce the p-eIF2α and the expression of GADD34 (Figure 1A,B). Consistent with previous studies, SARS2-N protein suppressed eIF2α phosphorylation which was triggered by poly I:C (Figure 1A,B). Induction of GADD34 is a primary event in the establishment of the host antiviral response to viral infection [38]. Of particular interest to us, the SARS2-N protein significantly downregulated the poly I:C-induced GADD34 protein (Figure 1A,B). By what mechanism does SARS2-N inhibit GADD34 expression? We next analyzed the effect of SARS2-N on the mRNA level of GADD34 and the turnover of the GADD34 protein by MG132 treatment. As shown in Figure 1C–E, SARS2-N had little effect on poly I:C-induced *GADD34* mRNA expression (Figure 1C) and a weak effect on the turnover of the GADD34 protein (Figure 1D,E), indicating that SARS2-N-induced suppression of the GADD34 protein may occur at the translational level. We also noticed that N^+^foci were induced in the context of low p-eIF2α, in contrast to the assembly of dsRNA-induced tSGs, which depends on eIF2α phosphorylation [33].

The 5′-UTR of human *GADD34* mRNA contains two non-overlapping upstream open reading frames (uORFs), uORF1 and uORF2. uORF2 contributes significantly to translational suppression of *GADD34* mRNA in basal non-stressed conditions, which is relieved by the phosphorylation of eIF2α upon stress [44]. To examine whether SARS2-N influences the translation of *GADD34* mRNA, we inserted *GADD34* 5′-UTR into a luciferase reporter plasmid with SV40 promoter and individually mutated the AUG of the two uORFs into AUA. Our results showed that the *GADD34* 5′-UTR-driven luciferase activity was repressed under basal conditions but activated by poly I:C (Figure 1F). However, overexpression of the SARS2-N protein inhibited poly I:C-induced luciferase activity, indicating that dsRNA-induced translation of *GADD34* mRNA was impaired by the SARS2-N protein. Mutation of uORF1 barely affected luciferase activity in both conditions, while mutation of uORF2 derepressed translation of *GADD34* mRNA in the basal condition and lost responsiveness to poly I:C. Interestingly, SARS2-N not only inhibited the activity of *GADD34* 5′-UTR and the uORF1 mutant, but also exerted its inhibitory effect on the uORF2 mutant. These results suggest that the SARS2-N protein suppressed dsRNA-induced translation of *GADD34* mRNA independent of uORF2 and may employ an alternative mechanism to inhibit *GADD34* translation.

### 2.2. SARS2-N Protein Sequesters GADD34 mRNA into N^+^foci

**Figure 2:**
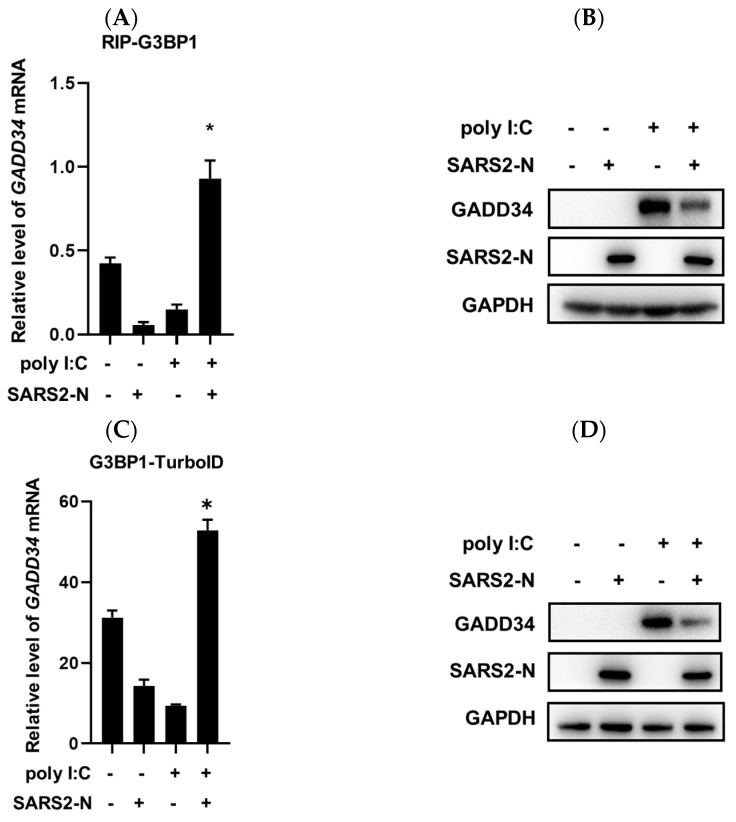

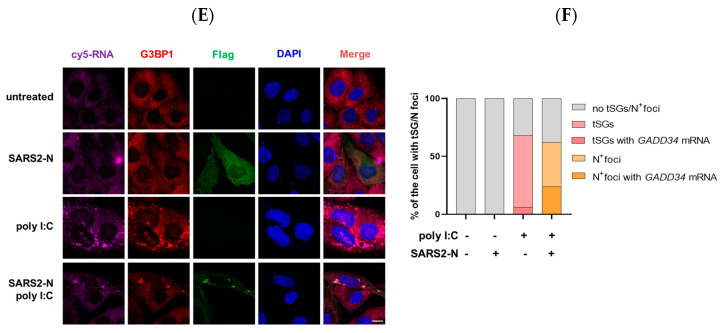
SARS2-N promotes the association of GADD34 mRNA with N^+^foci. (**A**) 16HBE cells were transfected with pCS2-Flag-SARS2-N and/or poly I:C followed by RIP with an anti-G3BP1 antibody. The enrichment of *GADD34* mRNA by G3BP1 was assessed by RT-qPCR and shown as the relative amount of *GADD34* mRNAs in immunoprecipitates compared to that of 5% input. Statistics: Student’s *t*-test (*, *p* < 0.05). (**B**) Cell lysates in the RIP assay were subjected to Western blot to detect GADD34, FLAG-SARS2-N, and GAPDH. (**C**) 16HBE cells were transfected with pCS2-Flag-SARS2-N and/or poly I:C together with pCS2-GFP-G3BP1-TurboID expression vector. Cells were harvested after the treatment of biotin for 15 min. mRNA associated with the biotinylated tSG protein or N^+^foci proteins were enriched with streptavidin beads followed by RT-qPCR for *GADD34* mRNA. Data are shown as the relative amount of *GADD34* mRNAs in streptavidin beads compared to that of 5% input. Statistics: Student’s *t*-test (*, *p* < 0.05). (**D**) Cell lysates in the TurboID assay were subjected to Western blot to detect GADD34, FLAG-SARS2-N, and GAPDH. (**E**) 16HBE cells were transfected with pCS2-Flag-SARS2-N and/or poly I:C together with cy5-labeled *GADD34* mRNA followed by immunostaining for Flag (green) and G3BP1 (red). In poly I:C-transfected cells, G3BP1^+^ condensates indicate tSGs. In SARS2-N and poly I:C-transfected cells, N^+^G3BP1^+^ condensates indicate N^+^foci. Scale bars: 20 μm. (**F**) Statistical analysis of the percentage of cells with tSGs or N^+^foci and the percentage of tSGs/N^+^foci that include *GADD34* mRNA is shown in panel (**E**). Data are shown as the mean ± SD (*n* = 3). Statistics: Student’s *t*-test.

We observed that tSGs were induced in poly I:C-treated 16HBE cells, while the SARS2-N protein remodeled tSGs to N^+^foci by hijacking G3BP1 [33]. As SGs are closely associated with translational regulation, we next tried to investigate whether G3BP1^+^ tSGs or N^+^foci are involved in SARS2-N-mediated inhibition of *GADD34* translation. First, we examined the interaction between *GADD34* mRNA and the G3BP1 protein using a G3BP1 antibody and performed RNA immunoprecipitation (RIP). As shown in Figure 2A, the recruitment of *GADD34* mRNA by G3BP1 was reduced upon poly I:C treatment to induce tSGs but increased by simultaneous overexpression of SARS2-N protein to induce N^+^foci. The changes in the enrichment of *GADD34* mRNA to G3BP1 inversely correlated to the changes in the GADD34 protein (Figure 2B). To verify this, we also overexpressed G3BP1-TurboID fusion protein and performed a TurboID proximity labeling assay. A comparable result was obtained as shown in Figure 2C,D. We next examined the subcellular localization of cy5-labeled *GADD34* mRNA and G3BP1 protein. Results of an immunofluorescence assay showed that the majority of *GADD34* mRNA was excluded from poly I:C-induced tSGs but co-localized with poly I:C and SARS2-N-induced N^+^foci (Figure 2E,F). Collectively, these results indicate that the SARS2-N protein promotes the interaction between *GADD34* mRNA and G3BP1 protein and isolates *GADD34* mRNA to N^+^foci, leading to the inhibition of *GADD34* expression.

### 2.3. SARS2-N Protein Inhibits GADD34 Expression to Attenuate Innate Immune Response

**Figure 3:**
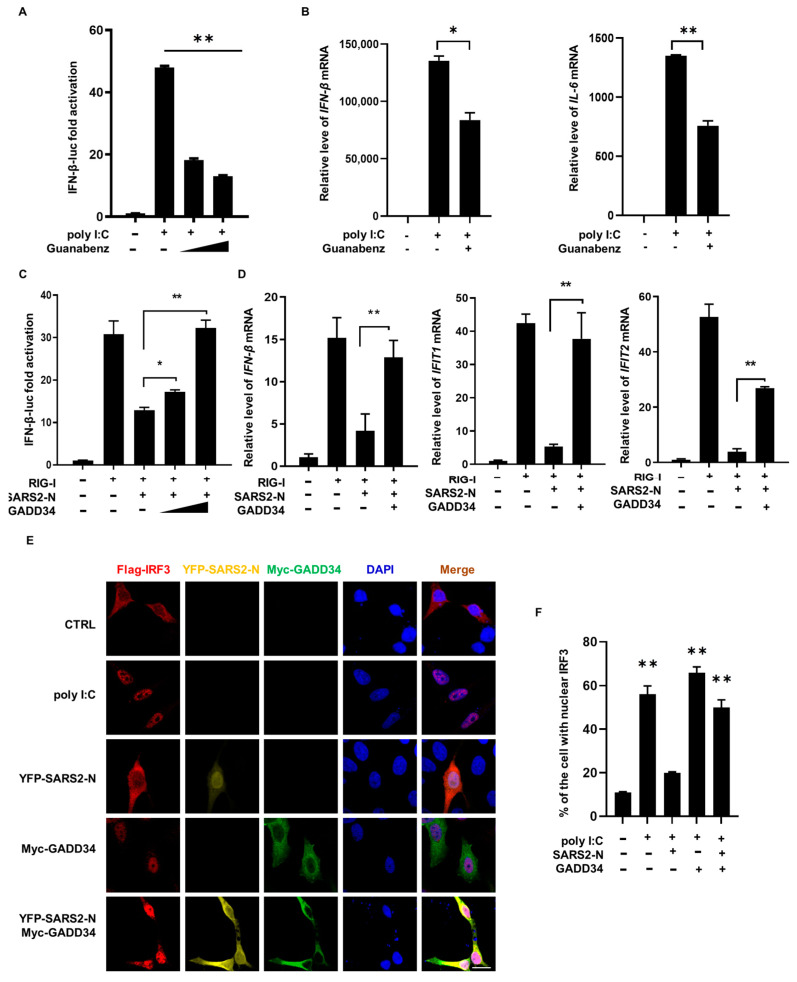
SARS2-N suppresses GADD34-mediated innate immunity response. (**A**) HEK293T cells were untreated or pretreated with 10 or 40 μM of Guanabenz for 8 h, then transfected with IFN-β reporter plasmids (IFN-β-Luc) and treated with poly I:C for 9 h followed by luciferase assays. Statistics: Student’s *t*-test (**, *p* < 0.01). (**B**) HEK293T cells were untreated, treated with poly I:C, or treated with poly I:C and 40 μM of Guanabenz for 9 h, followed by RT-qPCR for IFN-β, IL-6, and ACTB. Statistics: Student’s *t*-test (*, *p* < 0.05; **, *p* < 0.01). (**C**) HEK293T cells were transfected with IFN-β reporter plasmids (IFN-β-Luc) together with indicated plasmids (RIG-I, pCS2-Flag-SARS2-N, and Myc-GADD34), followed by luciferase assays. Statistics: Student’s *t*-test (*, *p* < 0.05; **, *p* < 0.01). (**D**) HEK293T cells were transfected with indicated plasmids (RIG-I, pCS2-Flag-SARS2-N, and Myc-GADD34) followed by RT-qPCR for IFN-β, IFIT1, IFIT2, and ACTB. Statistics: Student’s *t*-test (**, *p* < 0.01). (**E**) HEK293T cells were transfected with Flag-IRF3 together with pCS2-YFP-SARS2-N and pCS2-Myc-GADD34 expression vectors and treated with poly I:C for 9 h, followed by immunostaining for Flag (red) and Myc (green). Scale bars: 20 μm. (**F**) Statistical analysis of the percentage of cells with nuclear IRF3 is shown in panel (**E**). Data are shown as the mean ± SD (*n* = 3). Statistics: Student’s *t*-test (**, *p* < 0.01).

Studies have demonstrated that GADD34 plays a crucial role in the innate immune response, especially in the production of IFN-β to counteract viral infection. Consistently, we found that inhibition of GADD34 by a GADD34-specific inhibitor Guanabenz [39] blocked the poly I:C-induced IFN-β promoter activity and downregulated the levels of *IFN-β* and *IL-6* mRNAs (Figure 3A,B). Therefore, we next investigated the significance of SARS2-N-mediated suppression of GADD34 expression in the innate immune response. As shown in Figure 3C,D, SARS2-N protein significantly suppressed the promoter activity of IFN-β upon RIG-I treatment and downregulated the *IFNB1*, *IFIT1*, and *IFIT2* mRNA levels stimulated by RIG-I, but enforced expression of GADD34 overcame this inhibitory effect. (Figure 3C,D). This suggests that the SARS2-N protein might suppress GADD34 expression to attenuate the innate immune response (Figure 3C,D).

Since the production of IFN-β is primarily regulated by IRF3, we next analyzed whether SARS2-N could potentially disrupt the function of IRF3 by suppressing GADD34. The poly I:C-induced innate immune response was activated via translocation of IRF3 to the nucleus (Figure 3E,F). SARS2-N significantly inhibited the nuclear translocation of IRF3. Interestingly, the inhibition of IRF3 nuclear translocation by SARS2-N was markedly reversed by the overexpression of GADD34. Taken together, these results indicate that SARS2-N protein inhibited GADD34 expression, and GADD34-dependent activation of innate immune response was compromised consequently.

### 2.4. GADD34 Barely Affects SARS2-N Protein-Mediated Induction of N^+^foci

**Figure 4:**
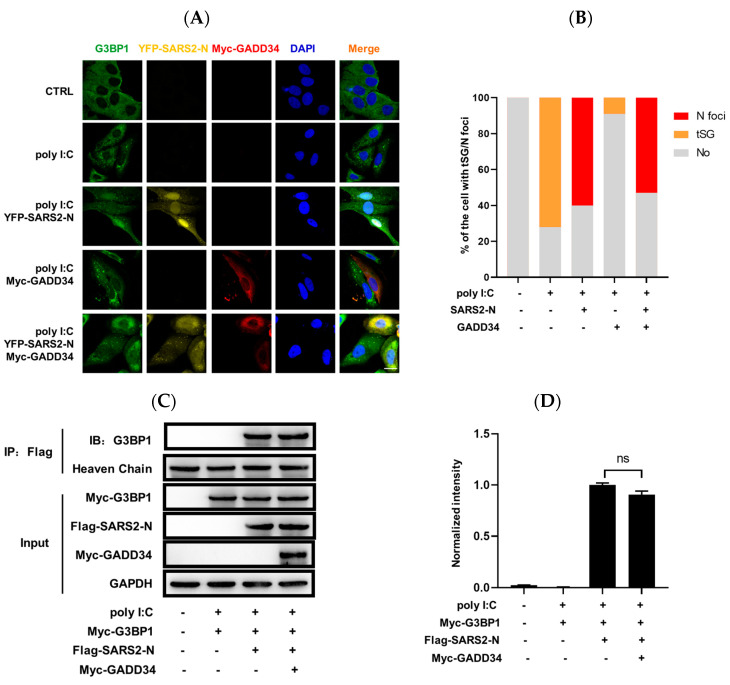
GADD34 barely affects SARS2-N protein-mediated induction of N^+^foci. (**A**) 16HBE cells were transfected with pCS2-YFP-SARS2-N and pCS2-Myc-GADD34 expression vectors and treated with poly I:C for 9 h, followed by immunostaining for G3BP1 (green) and Myc (red). Scale bars: 20 μm. (**B**) Statistical analysis of the percentage of cells with tSGs or N^+^foci is shown in panel A. Data are shown as the mean ± SD (*n* = 3). Statistics: Student’s *t*-test. (**C**) 16HBE cells transfected with pCS2-Myc-G3BP1, pCS2-Flag-SARS2-N, and pCS2-Myc-GADD34 were subjected to immunoprecipitation with an anti-Flag antibody. The presence of G3BP1 in the immunoprecipitates was assessed by Western blot against anti-G3BP1 antibodies. (**D**) Quantification of immunoblot intensities is shown in (**C**). The intensity is the ratio of the G3BP1 band to the GAPDH band. Bars indicate mean ± SD (*n* = 3). Statistical analysis was performed with Student’s *t*-test (ns, no significance).

G3BP1^+^ tSGs are usually formed in host cells in response to viral dsRNA to repress viral translation and stimulate the host’s innate immune response [18]. To investigate whether GADD34 could impact SG remodeling and restore innate immunity, we performed an immunofluorescence assay to detect SG formation in 16HBE cells. As shown in Figure 4A,B, tSGs were induced in about 72% of poly I:C treated cells. Consistent with our previous studies, the expression of SARS2-N significantly inhibited tSGs assembly but facilitated the formation of N^+^foci. Additionally, almost all GADD34-expressing cells lacked tSGs owing to its activity in eIF2α dephosphorylation and tSGs disassembly. Interestingly, when we co-expressed GADD34 with SARS2-N in 16HBE cells, we observed that 52% of these co-expressing cells maintained persistent N^+^foci, but no tSGs. This indicated that GADD34 could significantly impair poly I:C-induced tSGs assembly that is dependent on eIF2α phosphorylation, but barely affects the SARS2-N protein-mediated induction of N^+^foci that is independent of eIF2α phosphorylation.

We know that the SARS2-N protein switches tSGs to N^+^foci by reshaping the interactome of G3BP1. Furthermore, we investigated whether GADD34 affected the interaction between SARS2-N and G3BP1 using a co-immunoprecipitation assay. As shown in Figure 4C,D, the interaction between SARS2-N and G3BP1 was observed after the treatment of poly I:C, while GADD34 did not affect their interaction. Collectively, these findings suggest that GADD34 has a negligible influence on the formation of N^+^foci and acts independently of or downstream of SG remodeling events to modulate innate immunity.

### 2.5. GADD34 Counteracts SARS2-N Protein in Regulation of Innate Immunity Dependent on KVRF Motif

**Figure 5:**
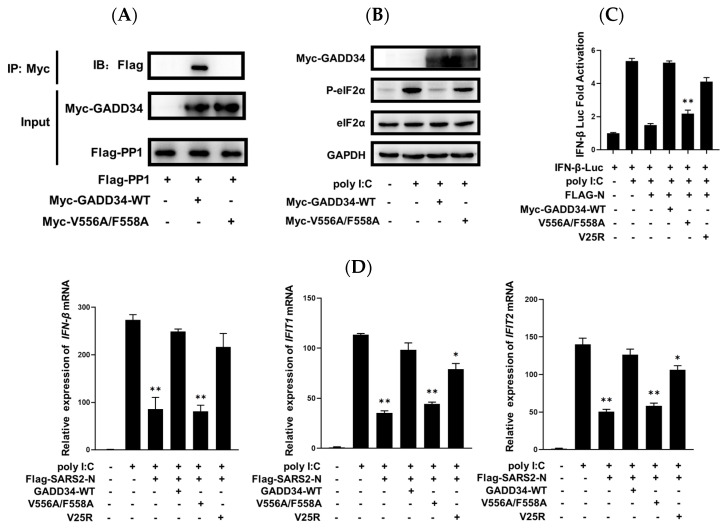

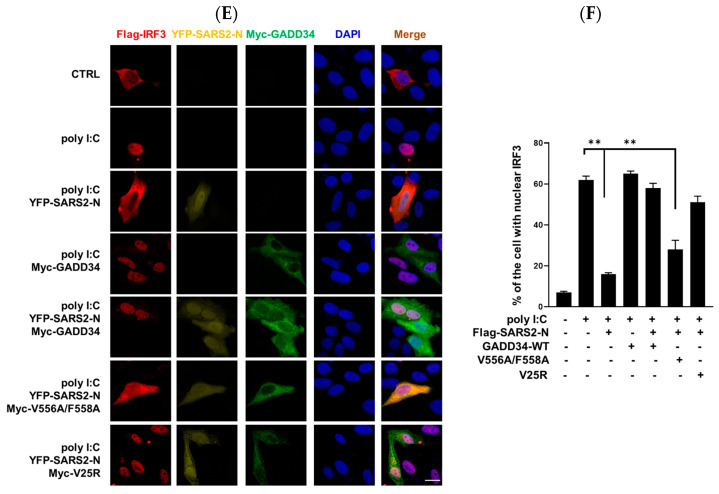
GADD34 rescues innate immunity suppressed by SARS2-N protein through KVRF motif. (**A**) HEK293T cells transfected with Flag-PP1c, Myc-GADD34 WT, or Myc-GADD34 V556A/F558 were subjected to immunoprecipitation with an anti-Myc antibody. The presence of PP1c in the immunoprecipitates was assessed by Western blot against anti-Flag antibodies. (**B**) HEK293T cells were transfected with Myc-GADD34 WT or Myc-GADD34 V556A/F558 and treated with poly I:C for the indicated time. Cell lysates were subjected to Western blot to detect eIF2α, p-eIF2α (S51), Myc-GADD34, and GAPDH. (**C**) HEK293T cells were transfected with IFN-β reporter plasmids (IFN-β-Luc) together with pCS2-Flag-SARS2-N and pCS2-Myc-GADD34 or its mutant expression vectors. After treating the cells with poly I:C for 9 h, a dual-luciferase assay was performed. Statistics: Student’s *t*-test (**, *p* < 0.01). (**D**) HEK293T cells were transfected with pCS2-Flag-SARS2-N and pCS2-Myc-GADD34 or its mutant expression vectors, and treated with poly I:C for 9 h, followed by RT-qPCR for *IFN-β*, *IFIT1*, *IFIT2,* and *18S RNA*. Statistics: Student’s *t*-test (*, *p* < 0.05; **, *p* < 0.01). (**E**) HEK293T cells were transfected with Flag-IRF3 together with pCS2-YFP-SARS2-N and pCS2-Myc-GADD34 or its mutant expression vectors, and treated with poly I:C for 9 h, followed by immunostaining for Flag (red) and Myc (green). Scale bars: 20 μm. (**F**) Statistical analysis of the percentage of cells with nuclear IRF3 is shown in panel (**E**). Data are shown as the mean ± SD (*n* = 3). Statistics: Student’s *t*-test (**, *p* < 0.01).

We next evaluated the potential mechanisms by which GADD34 counteracts the inhibition of innate immunity by SARS2-N protein. Previous studies showed that the GADD34 _555_KVRF_559_ motif binds with PP1 to drive its dephosphorylation activity [45], which is essential for cytokine production. We employed two mutants: V556A/F558A that is defective in interaction with PP1 and dephosphorylation of p-eIF2α (Figure 5A,B) and V25R that cannot localize to the endoplasmic reticulum (ER) but effectively scaffolds PP1 [45,46]. As shown in Figure 5C, the SARS2-N protein markedly suppressed the IFN-β promoter activation induced by poly I:C, whereas the expression of wild-type GADD34 completely counteracted the inhibitory effect of the SARS2-N protein. Similar to the wild-type of GADD34, V25R can also substantially rescue the promoter activity of IFN-β, which was suppressed by the SARS2-N protein. In contrast, overexpression of V556A/F558A barely affected the activity of the IFN-β promoter (Figure 5C). Moreover, the downregulation of *IFN-β*, *IFIT1*, and *IFIT2* mRNA levels by SARS2-N was also rescued by wild-type GADD34 or V25R, but not by V556A/F558A (Figure 5D). This observation suggests that the _555_KVRF_559_ is required for GADD34 to counteract SARS2-N-mediated suppression of innate immunity.

We have shown that GADD34 promotes the nuclear translocation of IRF3 (Figure 3E,F). We next performed immunofluorescence assays to investigate whether GADD34 regulates the nuclear translocation of IRF3 depending on the _555_KVRF_559_ motif. As illustrated in Figure 5E,F, SARS2-N protein significantly inhibited nuclear translocation of IRF3 induced by poly I:C. Either wild-type GADD34 or V25R effectively overcame these inhibitions by the SARS2-N protein, while V556A/F558A failed to recover the nuclear import of IRF3 (Figure 5E,F). Taken together, this indicates that GADD34 antagonizes SARS2-N protein-mediated suppression of innate immune response dependent on the _555_KVRF_559_ motif.

### 2.6. GADD34 Suppresses Viral Replication Facilitated by SARS2-N Protein

**Figure 6:**
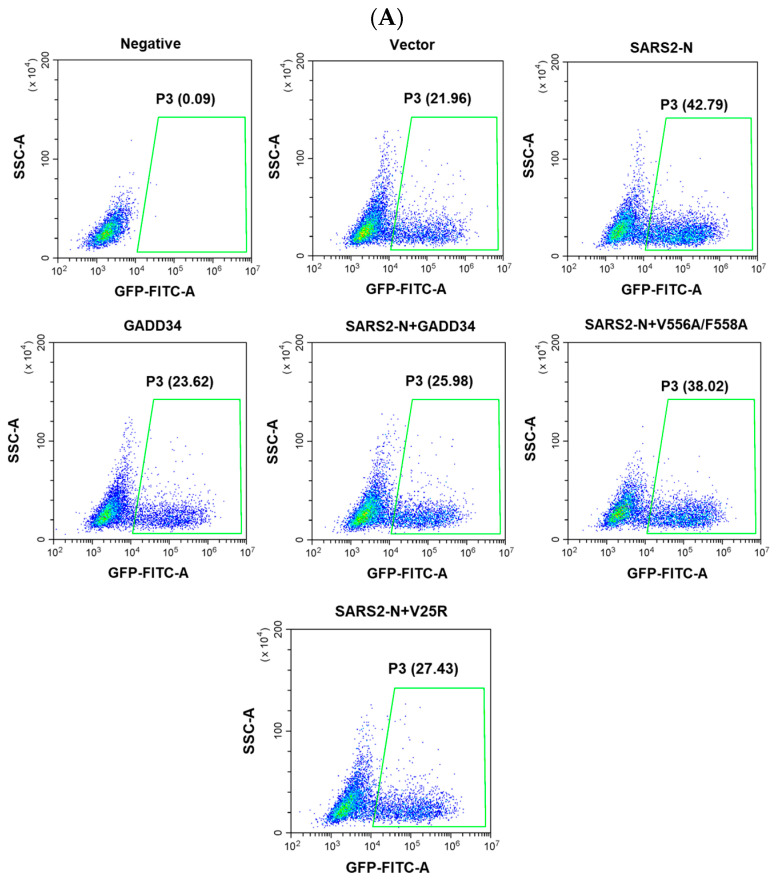

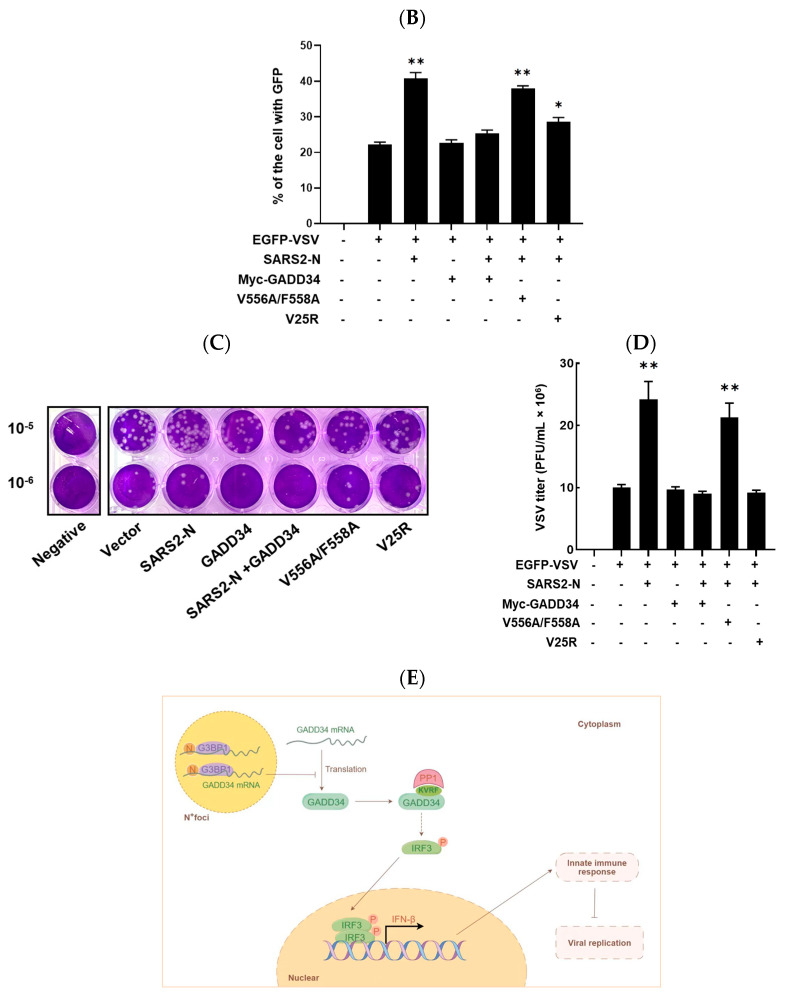
GADD34 suppresses viral replication mainly by KVRF motif. (**A**) HEK293T cells were transfected with plasmids as indicated. Thirty-six hours later, cells were infected with VSV-GFP (MOI of 0.001). Twelve hours after virus infection, cells were collected for flow cytometry to detect the rate of GFP^+^ cells and the supernatant was collected for the plaque assay. (**B**) Statistical analysis of the percentage of cells with VSV-GFP is shown in panel (**A**). The data are presented as the mean ± SD (*n* = 3). Statistics: Student’s *t*-test (*, *p* < 0.05 and **, *p* < 0.01). (**C**) Vero-E6 cells were plated in 24-well plates for culture prior to the experiment. When the cells reached 100% confluency, the collected supernatant was diluted by a 10-fold gradient for infection. After 24 h, the number of plaques was counted and the virus titer was calculated. (**D**) Statistical analysis of VSV titers is shown in panel (**C**). The data are presented as the mean ± SD (*n* = 3). Statistics: Student’s *t*-test (**, *p* < 0.01). (**E**) The proposed model that SARS2-N protein inhibits GADD34 expression and its physiological significance to the innate immune response and viral replication.

Our previous studies showed that SARS2-N facilitates viral replication by suppressing innate immunity [33]. Here, we utilized VSV-GFP as a model virus to investigate whether GADD34 may play a role in regulating viral replication through antagonizing the SARS2-N-mediated innate immunosuppression. Consistent with our previous studies, flow cytometry showed that the expression of SARS2-N markedly elevated the percentage of GFP-positive cells (Figure 6A,B). The expression of either the wild-type GADD34 or V25R mutant led to a notable decrease in the proportion of GFP-positive cells. However, the V556A/F558A mutant, which is deficient in inducing nuclear translocation of IRF3, failed to inhibit the replication of VSV-GFP, as there was no significant difference in the percentage of VSV-GFP in V556A/F558A-expressing cells compared to that in SARS2-N transfected cells. In addition, a viral plaque assay conducted in SARS-CoV-2-susceptible Vero-E6 cells showed that the VSV-GFP titer in the supernatant of cells expressing SARS2-N protein was significantly higher than that of the control group. Co-expression of wild-type GADD34 or V25R but not V556A/F558A reduced viral replication stimulated by the SARS2-N protein (Figure 6C,D). Collectively, these results demonstrate that GADD34 antagonized viral replication facilitated by the SARS2-N protein dependent on the KVRF motif, which may be due to the antagonistic action of the GADD34 and SARS2-N protein in the modulation of innate immunity.

## 3. Materials and Methods

### 3.1. Cell Culture

The human lung epithelial cell line 16HBE and the human embryonic kidney cell line HEK293T were grown in Dulbecco’s Modified Eagle Medium (DMEM; Gibco, Gaithersburg, MD, USA) supplemented with 10% fetal bovine serum (FBS; ExCell Bio, Shanghai, China) and 1% penicillin/streptomycin (Gibco, Gaithersburg, MD, USA) under standard tissue culture conditions (37 °C, 5% CO_2_).

### 3.2. Plasmids and Reagents

SARS2-N cDNA fragments were polymerase chain reaction (PCR)-amplified and subcloned into the pCS2 vector. hGADD34 protein-coding fragments were PCR-amplified from the cDNA of HEK293T and subcloned into the pCS2 vector. GADD34 expression plasmids with point mutation (V556A/F558A and V25R) were constructed with a Q5 Site-Directed Mutagenesis Kit (New England Biolabs, Ipswich, MA, USA). Luciferase reporter plasmids (GADD34 5′UTR-luc, GADD34 uORF1-AUA-luc, and GADD34 uORF2-AUA-luc) were kindly provided by Wentao Qiao of Nankai University, Tianjin, China. Guanabenz (APExBIO, Shanghai, China, #B1355) and MG132 (Meilun, Dalian, China, #MB5137) were used at concentrations of 40 μM and 20 μM, and treated for 9 h and 6 h, respectively.

### 3.3. Stress Treatment

Polyinosinic:polycytidylic acid (poly I:C; Sigma-Aldrich, St. Louis, MO, USA) was dissolved in RNase-free water containing 0.98% NaCl to make 2.5 mg/mL stock solution. Before use, the poly I:C was incubated at 50 °C for 20 min, followed by slow cooling to room temperature for annealing. To mimic stress induced by viruses, 2 μg/mL poly I:C was transfected into the cells with lipofectamine 2000 (Thermo Fisher, Waltham, MA, USA) for 6 h or the indicated time.

### 3.4. Immunofluorescence

HEK293T cells or 16HBE cells were seeded to reach 40%–60% confluence in 6-well plates with cover slips (Thermo Fisher, Waltham, MA, USA). After transfected with plasmids for 40 h, the cells were treated with poly I:C for 9 h to induce a stress response. The cells were fixed with 4% paraformaldehyde for 30 min at 4 °C and washed three times with phosphate-buffered saline (PBS). Next, the fixed cells were incubated in blocking buffer containing 1% bovine serum albumin (Sigma-Aldrich, St. Louis, MO, USA) and 1% normal goat serum (Jackson, West Grove, PA, USA) in PBS with 0.1% Triton X-100 for 30 min at room temperature. The cells were then incubated with primary antibodies overnight at 4 °C. The next day, the cells were washed three times with PBS and incubated with secondary antibodies conjugated to Alexa Fluor 488 or Alexa Fluor 594 (Thermo Fisher, Waltham, MA, USA) for 1 h at room temperature and kept in a dark environment. The nuclei were stained with DAPI (Sigma-Aldrich, St. Louis, MO, USA). Cells were then washed three times in PBS for 10 min each time. Images were acquired with a Zeiss LSM880 confocal microscope. The following antibodies were used: Flag (Sigma-Aldrich, St. Louis, MO, USA, #F1804), Myc (Sigma-Aldrich, St. Louis, MO, USA, #M5546), and G3BP1 (Santa Cruz Biotechnology, Dallas, TX, USA, #81940).

### 3.5. Luciferase Assay

HEK293T cells or 16HBE cells were seeded into 24-well plates at 1.5  ×  10^5^ cells per well. After 24 h, the cells were transfected with the indicated luciferase reporter plasmid, alongside the pGL3–SV40–Luc as the internal control and the empty expression vector to normalize the DNA dose. The cells were cultured for 40 h after transfection, and then stimulated with 2 μg/mL poly I:C for 9 h. Then the cells were collected, and luciferase activity was measured using the Dual-Luciferase Reporter Assay System (Promega, Madison, WI, USA). Luciferase values were normalized to Renilla luciferase and are expressed as the mean ± standard deviation (SD) of three sets of experiments.

### 3.6. Reverse Transcription Quantitative Polymerase Chain Reaction (RT-qPCR)

Total RNA was extracted using TRIzol Reagent (Thermo Fisher) as previously described [47]. cDNA was synthesized using the SuperScript III first-strand synthesis system (Thermo Fisher). Real-time qPCR was performed using the Power SYBR Green PCR Master Mix and StepOnePlus Real-Time PCR System (Thermo Fisher). The normalized value in each sample was derived from the relative quantity of target mRNA divided by the relative quantity of *18S rRNA* or *ACTB* (encoding β-actin). The relative mRNA expression level was derived from the threshold cycle (2^ΔΔC^T) by use of the comparative *C*_T_ method. Primers used in this study are shown in Supporting Information: Table S1.

### 3.7. Immunoprecipitation and Immunoblotting

The 16HBE cells were transfected with the indicated plasmids using Lipofectamine 2000 Reagent (Thermo Fisher). After being transfected for 40 h, the cells were treated with poly I:C for 9 h. Coimmunoprecipitation was performed as described previously [48]. Tagged proteins were isolated by immunoprecipitation with anti-Myc (Sigma-Aldrich) or anti-FLAG (Sigma-Aldrich) antibodies and Protein A/G beads (Smart Lifesciences, Changzhou, China). The protein levels were analyzed by immunoblotting as described previously [48]. Other antibodies used were as follows: anti-GADD34 (Proteintech, Wuhan, China, #10449-1-AP), anti-eIF2α (Santa Cruz Biotechnology, #sc-133132), anti-phospho-eIF2α (Cell Signaling Technology, Danvers, MA, USA, #9721S), and anti-GAPDH (Proteintech, #60004-1-Ig).

### 3.8. RNA Immunoprecipitation (RIP)

RIP was performed as described previously [49]. After 16HBE cells were transfected with expression plasmids for 40 h, the cells were treated with poly I:C for 9 h. Then, the cells were washed twice with cold PBS and lysed in lysis buffer for 10 min at 4 °C. Cell lysates were centrifuged at 4 °C at 14,000 rpm for 10 min. A total of 5% supernatant was used as input, and the rest of the supernatant was incubated with a G3BP1 antibody (Santa Cruz Biotechnology) and rotated overnight at 4 °C. Protein A/G beads (Smart Lifesciences) were added to the above solution, and then rotated at 4 °C for 3 h. After centrifuging at 3400 rpm for 1 min, the supernatant was discarded, and the beads were washed four times with RIP buffer. Finally, RNAs were extracted from the beads by Trizol (Thermo Fisher) and used for RT-qPCR. The enrichment of *GADD34* mRNA by G3BP1 was assessed by comparing the relative amount of *GADD34* mRNA in immunoprecipitates to that of 5% input.

### 3.9. TurboID Proximity Labeling Assay

A TurboID was performed as described previously [33]. 16HBE cells were seeded in 6 cm dishes. After 24 h, the cells were transfected with pCS2–GFP–G3BP1–TurboID plasmid with or without the SARS2-N protein expression plasmid. A total of 40 h after transfection, the cells were treated with poly I:C for 9 h to induce a stress response. Before harvesting, the cells were treated with 0.5 mM biotin (Sangon Biotech, Shanghai, China) for 15 min. Cell collection and lysis were performed as previously described [33]. Cell lysates were centrifuged at 14,000 rpm for 5 min at 4 °C, 20 μL of supernatant was used as input, and the remaining supernatant was incubated with 40 μL of streptavidin beads (Smart Lifesciences) overnight at 4 °C. The streptavidin beads were washed three times with lysis buffer. mRNAs associated with the biotinylated tSG protein or N^+^foci proteins were extracted from the streptavidin beads by Trizol (Thermo Fisher) and used for RT-qPCR. The enrichment of *GADD34* mRNA was assessed by comparing the relative amount of *GADD34* mRNA in streptavidin beads to that of 5% input.

### 3.10. Viral Infection and Flow Cytometry Analysis

GFP-labeled vesicular stomatitis virus (VSV-GFP, kindly provided by Peihui Wang, Shandong University, China) was used to infect HEK293T cells as described previously [33]. A total of 36 h after transfection with the SARS2-N expression plasmid, the medium was discarded, and HEK293T cells were washed twice with serum-free DMEM. VSV-GFP was diluted to the desired multiplicity of infection (MOI) with serum-free DMEM and used to infect HEK293T cells for 1 h. After infection, the virus–medium complex was replaced with DMEM containing 10% FBS. After 12 h, HEK293T cells were harvested by trypsinization for flow cytometry to determine the rate of GFP^+^ cells (no less than 10,000 cells in each group).

### 3.11. Viral Plaque Assays

HEK293T cells were infected with VSV-GFP as described above [33]. 12 h after viral infection, HEK293T cell supernatants were collected to infect Vero-E6 cells for the viral plaque assay. The day before infection, Vero-E6 cells were plated in 24-well plates. After reaching 100% confluence, the collected supernatant was diluted with a 10-fold gradient (10^−1^, 10^−2^ … 10^−7^), and Vero-E6 cells were infected for 30 min. The supernatant was discarded, and the cells were covered with DMEM containing 0.5% agar and 2% FBS. After the mixture was completely solidified, the cells were incubated for 24 h before fixation with paraformaldehyde for 30 min. Virus titers were calculated by discarding the agar–medium mixture and subsequently staining with 0.05% crystal violet to count the number of plaques on the cell monolayer.

### 3.12. In Vitro Transcription

The pCS2+ plasmid containing the GADD34 5′-UTR and coding sequence was linearized and used as a template for in vitro transcription. Cy5-labeled *GADD34* mRNA was transcribed using HyperScribe™ SP6 High Yield RNA Synthesis Kit and Cy5-UTP (APExBIO, Shanghai, China) according to the manufacturer’s instructions.

### 3.13. Statistical Analyses

Statistical analyses were performed with GraphPad Prism. All results are expressed as the mean ± SD of at least three independent biological replicates. The statistical significance of differences between different groups was determined using the unpaired two-tailed Student’s t test. Significance was assumed for * *p* < 0.05 and ** *p* < 0.01.

## 4. Discussion

In our study, we were the first to report that the SARS-CoV-2 N protein hijacks the cellular antiviral stress granules to assemble N^+^foci, which inhibits GADD34 and, in turn, inhibits the nuclear–cytoplasmic shuttling of IRF3. We provided insight into a novel mechanism by which SARS-CoV-2 suppresses IRF3 via N^+^foci.

During viral infection, the host’s PKR is activated by viral dsRNA, leading to the phosphorylation of eIF2α and the formation of SGs [26], which limits viral infection by blocking viral translation and sequestering host factors required for viral replication [50]. This stress response rapidly inhibits the synthesis of viral proteins prior to the upregulation of host antiviral proteins, serving as a crucial host innate immune defense. However, most viruses have evolved strategies to counteract this innate immunity [51]. Multiple viruses evade the sequestration of viral factors into SGs by blocking the formation of tSGs [52,53]. Alternatively, some viruses induce the formation of aSGs by redirecting the core proteins of stress granules [24,25,26]. Previous reports showed that viruses may induce the formation of aSGs and recruit host translation machinery, assembling viral factories that facilitate their replication [28,29,30,31]. Our previous studies found that the N protein of SARS-CoV-2 hijacks G3BP1, remodeling tSGs and inducing the formation of N^+^foci, which suppresses the expression of IFN-β and IL-6 [32,33]. However, how N^+^foci impair the host’s innate immunity remains unclear. In this study, we found that the SARS2-N protein inhibits dsRNA-induced expression of GADD34. The SARS2-N protein sequesters *GADD34* mRNA into N^+^foci, thereby suppressing the expression of GADD34 and subsequent nuclear translocation of IRF3. Thus, our findings reveal a novel GADD34-dependent mechanism by which SARS-CoV-2 suppresses innate immunity (Figure 6E).

GADD34 is a classic stress-responsive protein that contributes to the ISR [35,54]. GADD34 participates in various biological processes through the association with PP1 [35]. For instance, the GADD34–PP1 complex induces the dephosphorylation of eIF2α, which in turn restores the translational homeostasis of certain proteins under cellular stress conditions [55]. Also, GADD34 is involved in the innate immune response by activating RIG-I and MDA5 through the phosphatase activity of PP1 [42]. In mouse embryonic fibroblasts (MEFs), the GADD34-PP1 complex was found to inhibit the mTOR signaling pathway by dephosphorylating TSC2 [56,57]. It has been reported that viruses can inhibit the phosphatase activity of GADD34, which benefits their replication. For example, in the cells infected with Human T-cell leukemia virus type 1 (HTLV-1), the viral protein HBZ is exported from the nucleus to the cytoplasm, where it interacts with GADD34 and subsequently activates the mTOR signaling pathway, thereby promoting viral replication [41]. In addition, the Measles virus (MV) inhibits the phosphatase activity of GADD34-PP1 by binding to DC-SIGN on the cell surface, which in turn prevents the dephosphorylation activation of both RIG-I and MDA5 and ultimately suppresses the IFN-I [42].

Nevertheless, to date, there is no evidence that viruses can directly modulate the expression of GADD34. However, our study shows that the SARS2-N protein directly inhibits the translation of *GADD34* mRNA. GADD34 is extremely short-lived due to proteasomal degradation [46,58], which only allows for the rapid production of local cytokines [35,59]. In contrast, the levels of *GADD34* mRNA remain high after stress release, which allows GADD34 to be rapidly translated in response to increased p-eIF2α levels when cells encounter subsequent or sustained stress. Consistent with previous reports, we discovered in this study that GADD34 undergoes proteasomal degradation in both dsRNA-treated and untreated cells. In addition, treatment with poly I:C significantly upregulates levels of *GADD34* mRNA. However, in the presence of SARS2-N protein, *GADD34* mRNA potentially interacted with G3BP1 and was sequestered to N^+^foci, leading to suppression of *GADD34* translation. This suggests that the SARS2-N protein also inhibits the cellular events associated with GADD34, such as innate immunity in response to viral infection.

Viral dsRNA-dependent eIF2α phosphorylation is accompanied by global suppression of mRNA translation and the induction of IFN-I. IFN-I functions to limit the spread of viruses, delaying viral replication due to the halting of protein synthesis. Notably, IFNs are efficiently translated during global protein synthesis inhibition, and the inductions of IFNs are dependent on GADD34 [38]. This highlights the crucial role of GADD34 in the host response to viral infection. Evidence has shown that GADD34 is engaged in the antivirus response. In MEF cells, GADD34 has been observed to suppress the replication of the Vesicular Stomatitis Virus (VSV) [56]. Furthermore, Clavarino et al. reported that GADD34 is necessary for the production of IFN-β in response to Chikungunya Virus (CHIKV) infection [37]. Additionally, the GADD34-deficient cells display heightened susceptibility to CHIKVs. Nevertheless, the mechanisms by which GADD34 participates in the host’s antiviral immune response are still poorly understood. In this study, we were surprised to find that GADD34 plays a crucial role in innate immunity by mediating the nuclear import of IRF3. The expression of GADD34 promotes the nuclear import of IRF3, thereby rescuing the cellular innate immune response that was suppressed by the SARS2-N protein. However, our study did not clarify how GADD34 mediates IRF3 nuclear translocation. Further investigation is required to fully elucidate this mechanism. In addition, previous studies have demonstrated that innate immune sensors and signal transduction factors are subject to strict post-translational modifications. For example, RIG-I- and MDA5-mediated signaling is regulated by ubiquitination [60,61] and phosphorylation [62,63]. Notably, the phosphatases PP1α and PP1γ have been identified as pivotal activators for both RIG-I and MDA5 signal transduction [42,64]. This may be the underlying mechanism by which GADD34 utilizes the phosphatase activity of PP1 to participate in immune signal regulation.

SARS-CoV-2 developed multiple strategies to antagonize cellular innate immunity, thereby facilitating viral replication. For example, the SARS-CoV-2 structural protein, spike (S), interacts with IRF3 and promotes proteasome-dependent degradation of IRF3 [8]. The SARS-CoV-2 membrane protein binds with MDA5 and TBK1, leading to the degradation of TBK1 through K48-linked ubiquitination, inhibiting IRF3 phosphorylation [9]. Similarly, the nonstructural proteins 6 (Nsp6) and Nsp13 of SARS-CoV-2 could interact with TBK1 and induce the phosphorylation of TBK1, consequently suppressing the activation of IRF3 [13,14]. Moreover, the Nsp3 cleaves IRF3 to attenuate the production of IFN-1 [65]. In contrast, Nsp5 antagonizes IFN production by retaining phosphorylated IRF3 in the cytoplasm, and it does not affect the homeostasis and phosphorylation of IRF3 [12]. Some accessory proteins, such as SARS-CoV-2 open reading frame 6 (ORF6), bind to the karyopherin subunit alpha-2 (KPNA2) to inhibit the nuclear translocation of IRF3 [11]. ORF9b interacts with RIG-I, MDA-5, MAVS, and TBK1, impeding the phosphorylation and nuclear translocation of IRF3 [15]. The above studies suggest that SARS-CoV-2 modulates innate immunity through diverse mechanisms, potentially influencing various stages of viral infection and disease progression. The extensive suppression of the host’s antiviral response may facilitate viral replication and infection.

In conclusion, we have shown a novel mechanism whereby the SARS2-N protein sequesters *GADD34* mRNA into N^+^foci which significantly repress the expression of GADD34 and thereby block GADD34-mediated IRF3 nuclear transport, leading to the suppression of innate immunity. SARS-CoV-2 may take advantage of these mechanisms to promote viral pathogenesis.

## Data Availability

All relevant data are within the manuscript and its Supporting Information files.

## Supplementary Materials

The following supporting information can be downloaded at: https://www.mdpi.com/article/10.3390/molecules29204792/s1, Table S1. List of primers for quantitative PCR.
