# Supplementary material for: SARS-CoV-2 Nucleocapsid Protein Antagonizes GADD34-Mediated Innate Immune Pathway through Atypical Foci
Source: Molecules. 2024 Oct 10;29(20):4792. doi: 10.3390/molecules29204792 (PMC11510332; doi:10.3390/molecules29204792)
Supplement: Supplementary file 1 [file molecules-29-04792-s001.zip › molecules-3182516-supplementary.pdf]

Supplementary table S1. List of primers for quantitative PCR

Reaction	Target	Sequence
quantitative PCR	*GADD34*	F: TGAGGCAGCCGGAGATAC<br>R: GTAGCCTGATGGGGTGCTT
	IFN-β	F: ATGACCAACAAGTGTCTCCTCC<br>R: GGAATCCAAGCAAGTTGTAGCTC
	IFIT1	F: TTGATGACGATGAAATGCCTGA<br>R: CAGGTCACCAGACTCCTCAC
	IFIT2	F: AAGCACCTCAAAGGGCAAAAC<br>R: TCGGCCCATGTGATAGTAGAC
	IL-6	F: ATGAACTCCTTCTCCACAAGCGC<br>R: GAAGAGCCCTCAGGCTGGACTG
	18S	F: AACCCGTTGAACCCCATT<br>R: CCATCCAATCGGTAGTAGCG
	ACTB	F: AGCGGGAAATCGTGCGTGAC<br>R: CAATGGTGATGACCTGGCCGT
